# A retrospective study of maxillofacial fractures among adult population in Malda, India

**DOI:** 10.5249/jivr.v17i1.1925

**Published:** 2025-01

**Authors:** Santanu Mukhopadhyay, Anjan Jana, Nikita Singhal

**Affiliations:** ^*a*^ Department of Dentistry, Malda Medical College and Hospital Malda West Bengal, India.

**Keywords:** Facial fractures, Maxillofacial trauma, Epidemiology

## Abstract

**Background:**

Maxillofacial injuries are common in emergency departments of hospitals. However, there is limited regional data on facial fracture patterns in rural India. This study examines the etiology and anatomical distribution of facial fractures among adults attending Malda Medical College and Hospital in eastern India.

**Methods:**

This retrospective study included all adult patients (≥18 years) with radiologically confirmed facial fractures from August 2018 to July 2023. Data were collected on demographics, etiology, and fracture location. Patients with isolated soft tissue injuries or dentoalveolar fractures were excluded. Data analysis was performed using Epi Info 7 software.

**Results:**

In this study, 387 individuals with 557 facial fractures (averaging 1.43 fractures per person) participated. The average age was 37.56 ± 3.80 years, featuring a higher proportion of males (77.8%). Road traffic accidents (RTAs) accounted for 60.5% of the cases, making them the primary factor. Falls came next at 18.6%, followed by violence (12.1%) and sports injuries (8.8%). The most commonly affected areas were the mandible (33.0%), zygomatic complex (28.2%), and nasal bones (11.8%). Mandibular fractures were primarily caused by RTAs in males, while falls were more common among females. The etiology of fractures varies significantly between males and females (p = 0.000).

**Conclusions:**

RTAs were the main reason for facial fractures in eastern India, predominantly affecting young males.

## Introduction

Patients with orofacial trauma are common occurrences in hospital emergency departments. According to reports, approximately 60% of patients with trauma suffer from maxillofacial injuries.^1-3^ The presentation of such injuries varies, ranging from isolated soft tissue injuries or dentoalveolar fractures to facial fractures involving multiple facial bones. Fractures of the facial bones often impact an individual’s function and appearance, necessitating the involvement of various medical specialties for proper treatment. Because of the closeness of crucial structures like the brain, eyes, and airway, maxillofacial trauma frequently results in life-threatening conditions.

Studies indicate that men are more frequently impacted by maxillofacial injuries compared to women, with younger age groups being particularly vulnerable.^4-10^ Road traffic accidents (RTAs), falls from height, injuries related to sports, and violence are the primary causes of orofacial trauma.^11-19^

Facial fracture patterns differ across populations due to differences in factors like geographic location, cultural practices, and socioeconomic conditions. In India, where the burden of road traffic accidents remains high, facial fractures are common and often need specialized care. However, the epidemiology of these injuries can differ significantly across different regions of the country.

Malda Medical College, a tertiary care center in West Bengal, India. This institute serves a predominantly rural and semi-urban population of Malda and adjacent districts of West Bengal. Patients with orofacial trauma from neighboring states of Jharkhand and Bihar also report to this institute for treatment. Facial fractures' patterns and causes in this region might contrast with those observed in other parts of India and globally. Therefore, this study aimed to explore the causes and locations of facial injuries among individuals attending this tertiary care center in eastern India

## Methods 

**Objectives and Specific Aims**

The primary objective of this study was to assess the epidemiology, etiology, and patterns of facial fractures among adults presenting to the Outpatient Dental Department at Malda Medical College and Hospital. The specific aims were:

To identify the demographic characteristics of patients with facial fractures.

To analyze the etiological factors leading to facial fractures.

To categorize the types and sites of facial fractures.

After obtaining approval from the Ethics Committee of Malda Medical College and Hospital (Reference No.: P/MLD-MC/IEC-23/119, date 25.8.23) this study was performed in accordance with the ethical standards of Malda Medical College and Hospital. Patient confidentiality was maintained by anonymizing data during analysis.

**Study Population**

This retrospective study was carried out in the Outpatient Dental Department at Malda Medical College and Hospital, focusing on facial fractures among adults of both genders. 

Adult patients, aged 18 years and above, presenting with facial fractures at Malda Medical College and Hospital between August 2018 and July 2023 were part of this study. Each patient underwent a CT scan of the face to confirm the diagnosis of facial fractures. Exclusion criteria:

Incomplete records

Patients below 18 years of age

Isolated soft tissue injuries

Dentoalveolar fractures

Information collected from hospital records included:

• Demographic Data: Age, gender

• Etiology of Fractures: Classified into road traffic accidents (RTA), falls, violence, and sports.

1) RTA includes automobile accidents, bike accidents, bicycle accidents, and E-rickshaw accidents of drivers, passengers, and pedestrians.

2) Falls from heights as well as from one’s own height

3) Violence

4) Sports- related injuries

 Facial fractures were categorized as mandibular, zygomatic, nasal, maxilla, orbital, panfacial, and Le Fort fractures. Mandibular fractures were further categorized into parasymphysis, angle, ramus, condyle, and coronoid. 

**Statistical Analysis**

Data analysis was performed using an Excel spreadsheet and Epi Info software (version 7, CDC, Atlanta, USA). We performed Chi-square and Fisher's exact tests to assess the association between predictor variables (age, gender, and etiology) and the outcome variable (type of fracture). We used descriptive statistics to analyze proportions, frequencies, and percentages. Chi-square tests were utilized to compare categorical variables, with p<0.05 considered statistically significant.

## Results

In this study, 387 patients with 557 facial fractures (averaging1.43 fractures per person) were documented. The ages of individuals ranged between 18 and 74 years (mean 37.56 ± 3.80 years). The study included 301 men (77.8%) and 86 women (22.2%), with a male-to-female ratio of 3.5:1. In the study sample, 139 patients (35.9%) fell within the 18-30 years age range. The remaining patients were distributed as follows: 98 subjects (25.3%) fell within the age bracket of 31-40 years, 68 participants (17.6%) were between the ages of 41 and 50 years, 50 (12.9%) were aged 51-60 years, and 32 patients (8.3%) were aged 61 years and older. 

**Etiology**

RTAs emerged as the primary factor behind facial injuries, leading to 234 cases (60.5%). This was followed by falls (72 cases, 18.6%), violence (47 cases, 12.1%), and sports-related injuries (34 cases, 8.8%). (Table 1). Dentoalveolar fractures were found in 80 patients (20.7%). The etiology of fractures varies significantly between males and females (p = 0.000).

**Table 1:** Distribution of Patients with Facial Fractures by Age, Gender, and Etiology

Age Group (Years)	Gender	Etiology				
		RTA	Falls	Violence	Sports	Total
**18–30**	Male	66 (61.7%)	10 (9.3%)	15 (14.0%)	16 (15.0%)	107 (100%)
	Female	10 (31.3%)	2 (6.3%)	7 (21.9%)	13 (40.6%)	32 (100%)
**31–40**	Male	53 (69.7%)	12 (15.8%)	10 (13.2%)	1 (1.3%)	76 (100%)
	Female	9 (40.9%)	5 (22.7%)	4 (18.2%)	4 (18.2%)	22 (100%)
**41–50**	Male	42 (79.2%)	5 (9.4%)	6 (11.3%)	0 (0.0%)	53 (100%)
	Female	9 (60.0%)	5 (33.3%)	1 (6.7%)	0 (0.0%)	15 (100%)
**51–60**	Male	30 (75.0%)	9 (22.5%)	1 (2.5%)	0 (0.0%)	40 (100%)
	Female	2 (20.0%)	7 (70.0%)	1 (10.0%)	0 (0.0%)	10 (100%)
**61 and Above**	Male	12 (48.0%)	11 (44.0%)	2 (8.0%)	0 (0.0%)	25 (100%)
	Female	1 (14.3%)	6 (85.7%)	0 (0.0%)	0 (0.0%)	7 (100%)
**Total**	Male	203 (67.4%)	47 (15.6%)	34 (11.3%)	17 (5.6%)	301 (100%)
	Female	31 (36.0%)	25 (29.1%)	13 (15.1%)	17 (19.8%)	86 (100%)
**Overall Total**	Both	234 (60.5%)	72 (18.6%)	47 (12.1%)	34 (8.8%)	387 (100%)

RTA= Road traffic accident Chi-Square Value (χ2) =38.4 p value=0.000 (statistically significant)

**Site of Fractures**

As shown in Table 2 , mandibular fractures were the most common, with 184 cases (33.0%). This was followed by zygomatic complex fractures (157 cases, 28.2%), nasal fractures (66 cases, 11.8%), and orbital fractures (51 cases, 9.2%). Maxillary fractures occurred in 49 cases (8.8%). Less common were panfacial fractures (13 cases, 2.3%), Le Fort 1 fractures (12 cases, 2.2%), Le Fort 2 fractures (17 cases, 3.1%), and Le Fort 3 fractures (8 cases, 1.4%). 

There was a highly significant association between the etiology and site of facial fractures (p<0.0000001), indicating that different causes of fractures significantly influence the types and locations of facial fractures.

**Table 2:** Site of Facial Fractures According to Etiologic Factors

Site	RTA	Falls	Sports	Violence	Total	Percent (%)
**Mandible**	88 (47.8%)	57 (31.0%)	26 (14.1%)	13 (7.1%)	184 (100%)	33.0
**Zygomatic**	108 (68.8%)	14 (8.9%)	5 (3.2%)	30 (19.1%)	157 (100%)	28.2
**Nasal**	34 (51.5%)	13 (19.7%)	10 (15.2%)	9 (13.6%)	66 (100%)	11.8
**Maxilla**	40 (81.6%)	4 (8.2%)	0 (0.0%)	5 (10.2%)	49 (100%)	8.8
**Panfacial**	13 (100%)	0 (0.0%)	0 (0.0%)	0 (0.0%)	13 (100%)	2.3
**Le Fort 1**	11 (91.7%)	1 (8.3%)	0 (0.0%)	0 (0.0%)	12 (100%)	2.2
**Le Fort 2**	16 (94.1%)	1 (5.9%)	0 (0.0%)	0 (0.0%)	17 (100%)	3.1
**Le Fort 3**	8 (100%)	0 (0.0%)	0 (0.0%)	0 (0.0%)	8 (100%)	1.4
**Overall total**	366 (65.7%)	92 (16.5%)	41 (7.4%)	58 (10.4%)	557 (100%)	100.0

RTA= Road traffic accident Chi-square=96.23, p value˂0.0000 (statistically significant)

**Mandibular Fractures**

RTAs were the leading factor behind fractures in the mandible among men in males (77 cases), while falls were more common in females (24 cases). Sports-related fractures were more frequent in females (15 cases) compared to males (11 cases). The differences in the causes of mandibular fractures between genders were statistically significant (p = 0.000, Chi-Square = 26.8) (Table 3).

**Table 3:** Patients with mandibular fractures according to etiology and gender

Gender	RTA	Fall	Violence	Sports	Total
**Male**	77 (43.5%)	33 (18.0%)	8 (4.3%)	11 (6.0%)	129 (70.1%)
**Female**	11 (6.0%)	24 (13.0%)	5 (2.7%)	15 (8.2%)	55 (29.9%)
**Total**	88 (47.8%)	57 (31.0%)	13 (7.1%)	26 (14.1%)	184 (100.0%)

RTA= Road traffic accident P=0.000, (statistically significant) Chi Square =26.8

**Site of Mandibular Fractures**

Table 4 shows the distribution of mandibular fractures by site and etiology, indicating that road traffic accidents (RTA) were the leading cause of fractures across most sites (47.5%). The Chi-square test (χ2 = 14.14, p = 0.292) shows no statistically significant association between the etiology and the site of mandibular fractures. 

**Table 4:** Distribution of Mandibular Fractures by Site and Etiology

Etiology	Parasymphysis	Angle	Condylar	Coronoid	Ramus	Total
**RTA**	45(51.7%)	37(48.7%)	24(39.3%)	6(100%)	6(100%)	118 (47.5%)
**Fall**	26(29.9%)	22(28.9%)	20(32.8%)	0(0%)	0(0%)	68 27.4%)
**Violence**	7(8.0%)	6(7.9%)	6(9.8%)	0(0%)	0(0%)	19 (7.6%)
**Sports**	9(10.3%)	11(14.5%)	11(18.0%)	0(0%)	0(0%)	32 (12.9%)
**Total**	87(100.0%)	76(100%)	61(100%)	6(100%)	6(100%)	236 (100.0%)

RTA= Road traffic accident Chi-square =14.14 p=0.292 no statistically significant association

## Discussion

Epidemiological characteristics of facial fractures differ among populations due to the influence of culture, geographic location, and patients’ socioeconomic conditions. Understanding the epidemiology of facial trauma is crucial for formulating preventive strategies and treatment plans specific to a particular region. In this research, the average patient age stood at 37.56 ± 3.80 years, with individuals in the 18–30-year bracket being predominantly impacted by facial trauma. These findings are comparable to reports from other studies.^4,5,8,11,16^ This trend might be due to individuals in their twenties and thirties being more engaged in social, work-related, and physical activities, which raises their exposure to injury.^1,4,14,15^ The average of 1.43 fractures per patient indicates a substantial burden of injury severity among the study population.

Our study reported a male predominance in facial fractures, with males being 3.5 times more commonly affected than females. This finding is consistent with existing literature on facial fractures.^15-28^ The higher incidence among men may be attributed to riskier behaviour and their more frequent involvement in dangerous occupations.

This research identified RTAs as the predominant cause of facial fractures. They accounted for over 60% of cases, a figure consistent with findings from other regions in India: 74.7% in Karnataka, 70% in Andhra Pradesh, 62.1% in Chennai, and 62% in Pondicherry.^6,7,21,22^

Similar findings have been observed worldwide. Sharifi et al. reviewed 32 studies from Iran and found that RTAs accounted for 68.97% of maxillofacial injuries.^16^ Likewise, a 2016 study identified RTAs as the leading cause (52% of facial injuries) in Brazil.^17^

Reports indicate that road accidents in India are on the rise. Common causes include speeding, driving on the wrong side, potholes (especially during the rainy season), drunk driving, mobile phone use, and a lack of awareness of traffic laws.^13^ Making seat belts compulsory for car passengers and helmets mandatory for motorcycle/scooter riders could help minimize RTAs. The prominence of RTAs as the primary reason for facial trauma highlights the necessity for enhanced road safety initiatives, such as tighter enforcement of traffic regulations, public awareness campaigns, and improved road infrastructure across India.

In the age bracket of 61 years and older, falls were the primary cause of maxillofacial injuries. These incidents accounted for 18.6% of the cases and were the second most frequent reason for injury. Several investigators also concluded that falls were the predominant cause of facial injuries.^19,23,26^ Dasukil et al. noted that falls are the most common reason for maxillofacial fractures among individuals under 10 and over 60 years of age.^29^ In younger age groups, men experience more facial fractures due to falls, while women are more commonly affected by falls in older age groups.^29^ Aging is associated with sensory impairments such as vision and hearing loss, cognitive and memory changes, as well as bone and muscular disorders, all of which increase the risk of falls.^29^

Several European investigations indicate that Violence or assaults predominantly lead to facial injuries.^1,4,23,24^ In contrast, violence and sports-related injuries were less frequent in this study and were mainly restricted to young adults, though they were still significant contributors to facial fractures. The lower prevalence of violence-related trauma compared to European studies may be due to cultural differences and lower rates of drug and alcohol use. Additionally, domestic violence is often underreported in many developing countries. Though sports-related injuries were less common, they highlight the need for protective gear and safety protocols in sports activities.

The present study indicates that the mandible represents the most frequently fractured facial bone, with the zygomatic bones following closely. These findings align with those of earlier studies.^2,3,4,8,9,12,26,28^ The mandible is more susceptible to fractures in maxillofacial injuries due to its prominent position and exposure. However, some studies have found the zygomatic bones,^5,17^ nasal bones,^10,27^ and orbital bones^14^ to be the main sites of facial fractures.

Among patients with RTAs as the cause, the zygomatic bone emerged as the most frequently fractured site, likely due to its anatomical location. RTAs often involve high-energy impacts that affect the facial skeleton, particularly the zygomatic bone. When a vehicle collides or hits an object, the face may come into contact with steering wheels, dashboards, or windshields, leading to zygomatic fractures. Trauma in this area can also result from blunt force impacts, such as those experienced in falls, sports, or physical altercations.

Regarding the distribution of mandibular fractures, the most common site varies across studies. Several investigators have identified the parasymphysis as the most common location, while Zuncar et al., Jariod Ferrer et al., and Aleksanyan et al. found the angle to be the most common site.^2,4,7,24,25,27,29^ Khandeparkar et al. identified the mandibular condyle as the predominant fracture location.^11^ Jariod Ferrer et al. suggested that the variation could be attributed to differences in the mechanism of injury.^24^

Dentoalveolar fractures are often underreported in maxillofacial injury studies, possibly because clinicians focus on more severe injuries. However, these fractures can impair chewing, speaking, and occlusion, and affect a patient's appearance. In this study, 20.7% of patients had dentoalveolar fractures, highlighting the vulnerability of this region in trauma, a rate higher compared to the results found by a Croatian study (8.9%) and an Indian study (6.6%).^4,11^

Our study on facial trauma in rural and semi-urban population of Malda, India, shows that road traffic accidents (RTAs) are the leading cause of injury, consistent with findings from other rural studies globally. For example, Malik et al. (2017) in India also identified RTAs as the primary cause of facial injuries, while Batista et al. (2012) in Brazil reported RTAs in urban areas but found animal-related accidents more common in rural areas.^30,31^ Similar to our study's male-to-female ratio of 3.5:1, studies by Ogunmuyiwa et al. (2015) in Nigeria and Cohn et al. (2020) in the USA also observed gender disparities in rural trauma.^32,33^ Mandibular fractures, found in 33% of our patients, were similarly the most common in Nigeria. These findings highlight the need for region-specific public health policies, considering local factors like road safety, violence, and socio-economic conditions.

Limitations of this research involve its retrospective nature and its single-center approach. Future research could benefit from multi-center studies, longer-term follow-ups, and investigations into the socioeconomic impacts of facial fractures. However, the findings of this study have several clinical implications. Understanding the common causes and sites of facial fractures can help create focused strategies for prevention and treatment. For instance, a high incidence of RTAs as a cause of facial fractures suggests that improving road safety and enforcing traffic regulations could significantly reduce the burden of these injuries.

## Conclusion

This study analyzed 387 patients with 557 facial fractures, identifying road traffic accidents (RTAs) as the primary cause, with falls, aggressive incidents or violence, and sports injuries also contributing. Fractures in the mandible and zygomatic bones were the most frequent, predominantly affecting males and the 18–30-year age group. The study also noted a significant prevalence of dentoalveolar fractures, often underreported but important due to their impact on function and aesthetics. These results show the necessity for enhanced traffic safety initiatives, public awareness, and the use of protective gear in both driving and sports.

**Acknowledgment:**

We would like to thank Prof (Dr) Partha Pratim Mukhopadhyay of Malda Medical College and Hospital for his help in this study.

**Authors’ Contributions:**

Santanu Mukhopadhyay: Study design, data collection, analysis and interpretation of data and writing.

Anjan Jana: Data collection, review of literature, writing and review of manuscript.

Nikita Singhal: Data collection, and writing of manuscript All authors have read and approved the final version of the manuscript.
